# G721-0282 inhibits cell growth and induces apoptosis in human osteosarcoma through down-regulation of the STAT3 pathway

**DOI:** 10.7150/ijbs.37781

**Published:** 2020-01-01

**Authors:** Kyung-Ran Park, Hyung-Mun Yun, Jin Tae Hong

**Affiliations:** 1 College of Pharmacy and Medical Research Center, Chungbuk National University, Chungbuk 194-31, Republic of Korea; 2 Department of Oral and Maxillofacial Pathology, School of Dentistry, Kyung Hee University, Seoul 02453, Republic of Korea.

**Keywords:** G721-0282, Chi3L1, STAT3, osteosarcoma, Src, MAPK

## Abstract

Osteosarcoma (OS) is considered the most common type of primary malignant bone tumor, which has an urgent need for more effective treatment. Recently, chitinase 3 like 1 (Chi3L1) expression has been found in a variety of cancer cells. However it is not known whether Chi3L1 regulates the STAT3 pathway in OS cells**.** Herein, we examined the effects of the G721-0282, a ligand of Chi3L1, *in vitro* and *in vivo* against OS cells. G721-0282 inhibited the proliferation of OS cells and induced apoptosis. This apoptosis was accompanied by upregulation of apoptotic proteins (PARP and procaspase-3), but downregulation of anti-apoptotic proteins (Survivin and Bcl-2). G721-0282 induced the inactivation of mitogen-activated protein kinases (MAPKs) with a decrease in the phosphorylation of Src and STAT3 in OS cells. Importantly, overexpression of Chi3L1 potentiated the effects of G721-0282, while knockdown of Chi3L1 attenuated the effects of G721-0282. Docking model study also showed that G721-0282 interacted with Chi3L1. In addition, G721-0282 inhibited cell migration, invasion, and colony formation. Furthermore, the anti-tumor effects of G721-0282 were observed in an xenograft *in vivo* model in association with the reduced expression of Chi3L1, PCNA, Cyclin D1, p-STAT3, as well as the increased expression of Chi3L1 was correlated with the p-STAT3 level in human bone tumor tissues. Taken together, a Chi3L1 ligand, G721-0282 may be an attractive therapeutic strategy for OS, especially *in vitro* and *in vivo* anti-proliferative effects against OS cells through the inhibition of the STAT3 pathway, and suggest the potentially therapeutic application of G721-0282 in the treatment of OS.

## Introduction

An osteosarcoma (OS) is a type of cancerous tumor that starts in the bone and most common type of sarcoma in children and adolescents 1. Consistent with its high incidence in adolescents, OS preferentially develops in the growth plates of the most rapidly growing bones such as the distal femur and proximal tibia 2. The survival rate for OS patients who undergo chemotherapy and surgery is very low to approximately 30 to 50 percent if the cancerous tumor tissues are successfully removed 3. Thus, the identification and development of novel molecules to effectively inhibit cell growth or induce apoptosis in OS are required.

Apoptosis is a biological process that is essential to all living organisms and is a key feature of tumor development 4-5. Caspase-3 activation leads to the degradation of cellular proteins to maintain cell survival and death, and regulated by the proapoptotic B-cell lymphoma protein-2 (Bcl2) family of proteins and the anti-apoptotic Bcl2 family of proteins. The signal transducers and activators of transcription 3 (STAT3) is constitutively activated in diverse tumors and also involved in tumor growth 6-8. Mitogen Activated Protein Kinases (MAPKs: ERK, p38, and JNK) signaling cascades is also important for full activation of STAT3 9-10. Several studies demonstrated that Src, a proto-oncogenic nonreceptor tyrosine kinase, activated STAT3, which resulted in the stimulation of tumor growth and the pathway plays important roles in tumorigenicity 11-13. Therefore, the identification and comprehensive understanding of novel molecules in the detailed roles of intracellular pathways in OS progression may represent promising therapeutic strategies for the treatment of patients with this malignancy. Recently, chitinase 3 like 1 (Chi3L1) expression has been found in a variety of cancer cells such as breast, lung, prostate, colon, rectum, ovary, kidney, glioblastomas, and malignant melanoma 14-20. We also previously selected 20 chemicals as candidate compounds for the ligand of Chi3L1 by using 3D chemical database analysis with X-ray structure-based virtual screening 21, and G721-0282 was obtained from ChemiDiv Inc. (http://www.chemdiv.com). In the present study, we investigated whether G721-0282, a candidate ligand of Chi3L1 regulates *in vitro* effects on proliferation, apoptosis, migration, invasion, and colony formation in OS cells, and *in vivo* anti-tumor effects in a xenograft mouse model. Our studies present the activity and the underlying mechanism of G721-0282 on the OS cells.

## Materials and Methods

### Animals

Mice were housed in standard cages in an Assessment and Accreditation of Laboratory Animal Care credited specific pathogen-free (SPF) animal facility on a 12h light-12h dark cycle. All protocols involving mice in this study were reviewed and approved by the Chungbuk National University Institutional Animal Care and Use Committee (IACUC) and complied with the Korean National Institute of Health Guide for the Care and Use of Laboratory Animals (CBNUA-792-15-01).

### G721-0282

G721-0282 (purity 98%) was purchased from ChemiDiv Inc. (San Diego, CA). Original solution was processed into a 200 ug/ml stock solution with dimethyl sulfoxide (DMSO).

### Cell culture and Transfection

MG63 and U2OS cells (human OS cell lines) were purchased from the Korean Cell Line Bank (Seoul, Korea) and were grown in Dulbecco's modified Eagle medium (DMEM) supplemented with 10% fetal bovine serum (FBS), penicillin (100 units/ml), and streptomycin (100 μg/mL) at 37 °C in a humidified atmosphere of 5% CO_2_ and 95% air. Direct seeding of cells (5 × 10^5^ cells/ 6 well plates) was performed for the experiments. After overnight incubation, culture medium was replaced with fresh DMEM and cells were treated with various concentration of G721-0282. For transfection, the cells were transfected using Lipofectamine 3000 or Lipofectamine RNAiMAX (Invitrogen) according to the manufacturer's instructions.

### Cell proliferation assay

Cell proliferation was measured by an 3-[4,5-dimethylthiazol-2-yl]-2,5-diphenyltetrazolium bromide (MTT) assay to detect NADH-dependent dehydrogenase activity as previously described 22.

### TUNEL assay

DNA fragmentation was examined by terminal deoxynucleotidyl transferase-mediated FITC-dUDP nick-end labeling (TUNEL). TUNEL assays were performed using the in situ Cell Death Detection Kit (Roche Diagnostics GmbH, Mannheim, Germany) according to the manufacturer's instructions.

### Annexin V assay

Annexin V assays were performed using the Annexin V Staining Kit (BioVision, Inc., Milpitas, CA) according to the manufacturer's instructions.

### Immunocytochemistry

Cells were grown on glass coverslips and incubated with G721-0282. Cells were fixed in 10% formalin for 15 min at room temperature. After washing three times in 1X PBS, the cells were permeabilized with 0.2% Triton X-100 in 1X PBS for 20 min, washed three times in 1X PBS, and then blocked with 5% BSA in 1X PBS for 1 h at room temperature. After then, the cells were incubated with anti-p-STAT3 (1:200, Cell signaling) antibody for overnight at room temperature, washed three times, and incubated with Alexa-488 conjugated secondary antibodies (1:500, Invitrogen, Carlsbad, CA) for 2 h at room temperature. The cells was stained with DAPI (Sigma-Aldrich) and washed three times, mounted on glass slides, and viewed on a confocal microscope (K1-Fluo Confocal Laser Scanning Microscope, Korea).

### Molecular docking model

Docking studies between G721-0282 and Chi3L1 were performed using Autodock VINA. Three-dimensional structures of the CHI3L1-DNA complexes were retrieved from the Protein Data Bank [PDB:1VKX], and a three-dimensional structure of CHI3L1 was built using Chem3D and ChemDraw, which was further prepared using AutodockTools. The grid box was centered on the CHI3L1 monomer, and the size of the grid box was adjusted to include the whole monomer. Docking experiments were performed at various default exhaustiveness values: 16, 24, 32, 40, and 60. Molecular graphics for the best binding model were generated using the Discovery Studio Visualizer.

### Immunohistochemistry

All specimens were fixed in formalin and embedded in paraffin for examination. Sections (5 μm thickness) were stained with hematoxylin and eosin (H&E) and analyzed by immunohistochemistry. The sections were deparaffinized by immersing into xylene solution, rehydrated, subjected to heat-mediated antigen retrieval treatment, washed with distilled water and proceed with immunohistochemical procedure. Endogenous peroxidase activity was quenched by incubation with 1% hydrogen peroxide solution in methanol for 30 min and washed with 1X PBS (Sigma, St. Louis, MO) for 5 min. Next, the sections were blocked with 5% BSA diluted in 1X PBS for 30 min, incubated overnight with specific antibodies at 4°C, and washed 3 times with 1X PBS. The immunological detection was started with incubation in horseradish peroxidase (HRP)-conjugated secondary antibodies (1:500, Jackson ImmunoResearch) for 1 h at room temperature. After washing with 1X PBS, chromogen development was performed with 0.02% 3, 3'-diaminobenzidine tetrahydrochloride (DAB, Vector Laboratories, Burlingame, CA) and slides counterstained with hematoxylin. Finally, sections were dehydrated with ethanol, cleared with xylene, and mounted with Permount (Fisher Scientific, Rockford, IL), and evaluated on a light microscope (Olympus, Tokyo, Japan).

### Soft agar assay

2 ml of 0.6% agar was layered in the bottom onto 6-well plates, followed by 3 ml of 0.3% agar as the top layer. MG63 cells were then plated with various concentrations of G721-0282 on the top layer. The cells were maintained at 37 °C in a humidified atmosphere of 5% CO_2_ and 95% air for 14 days, and the colonies were observed and quantified under a light microscope.

### Boyden chamber assay

The invasion assay was performed based on published methods with some modifications. Exponentially growing cells after 1 day of growth in complete medium were collected, and the old medium was saved as conditioned medium for chemotaxis. The Nuclepore filter was coated with 1 ml matrigel on ice, then incubated at 37 °C for 30 min. Boyden chamber was assembled with 24 μl of conditioned medium in each lower chamber, matrigel-coated Nuclepore filter facing upward, and 50 μl of serum-reduced (5% or less) medium containing 2 × 10^4^ cells in each upper chamber. After incubating the Boyden chamber in tissue culture incubator for 6 h, the filter was reversed, fixed in methanol, and stained by 0.5% crystal violet. The cells that have traveled past the filter were counted as invasive cells.

### Western blot analysis

Western blot analysis was performed as previously described 23.

### Xenograft animal model

Six-week-old male BALB/c athymic nude mice were purchased from Samtako (Osan, Kyoung Gi-Do, Korea). Human OS MG63 cells (1 × 10^8^ cells) were injected subcutaneously into the right-lower flanks of the carrier mice as previously described 24-25. After 10 days, two groups of mice (n = 5) were i.p. injected with G721-0282 (18.9 mg/kg) two times a week for 35 days. The control group of mice (n = 5) were treated with vehicle two times a week for 35 days. At the end of the experiment, cervical dislocation was performed for euthanasia.

### Human bone normal and tumor samples

Human bone normal and tumor tissue lysates were obtained from GeneTex, Inc (Irvine, CA) and analyzed by western blotting.

### Statistical analysis

The data were analyzed using the GraphPad Prism version 5 program (GraphPad Software, Inc., San Diego, CA). Data are presented as mean ± S.E.M. Satistical significance was performed on the data using Newman-Keuls test. A value of P < 0.05 was considered to be statistically significant.

## Results

### G721-0282 inhibits cell growth in osteosarcoma cells

**Figure 1:**
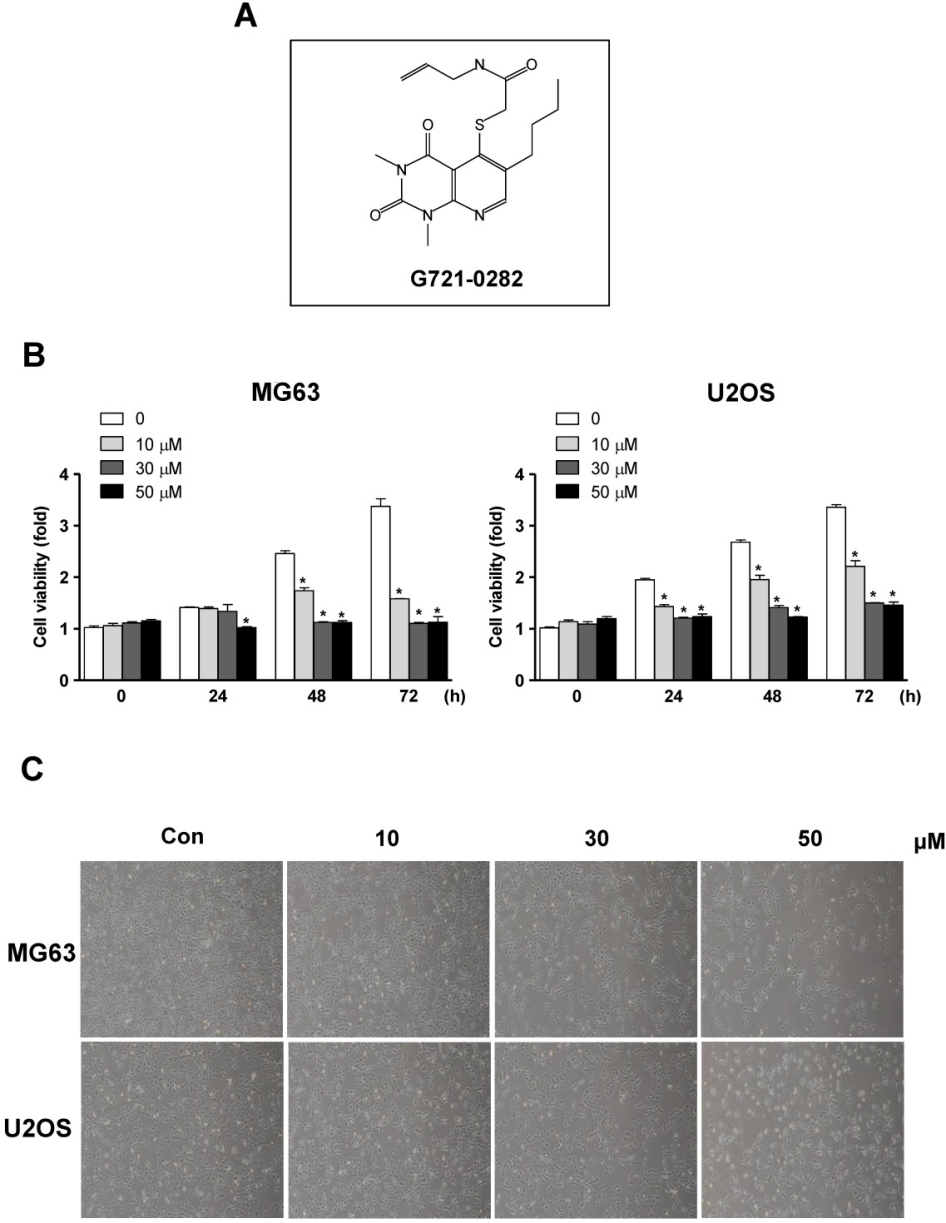
** Effects of G721-0282 on cell growth in osteosarcoma cells. (A)** Chemical structure of G721-0282. **(B)** After MG63 and U2OS (1 × 10^4^ cells/well) were seeded onto 96-well plates, the cells were cultured in the indicated concentration of G721-0282 for 24, 48, and 72 h. Cell proliferation of MG63 and U2OS was measured using MTT assay. **(C)** After treatment with G721-0282 for 24 h, the morphological changes of MG63 and U2OS were observed. The results are representative of three independent experiments. *: statistically significant difference compared to the control group (p<0.05).

To examine the activity of G721-0282 on cell proliferation on MG63 and U2OS cells, the cells were treated for 24 h, 48 h, and 72 h of G721-0282 with the indicated concentrations and then cell viability was analyzed. G721-0282 significantly suppressed cell proliferation in these cells in a dose and time dependent manner (Fig. 1B). Morphologic observation clearly showed that the MG63 cells and U2OS cells were gradually reduced in size and changed into a small-round-single cell shape by the treatment of G721-0282 in a dose dependent manner (Fig. 1C).

### G721-0282 induces apoptosis and cell cycle arrests

**Figure 2:**
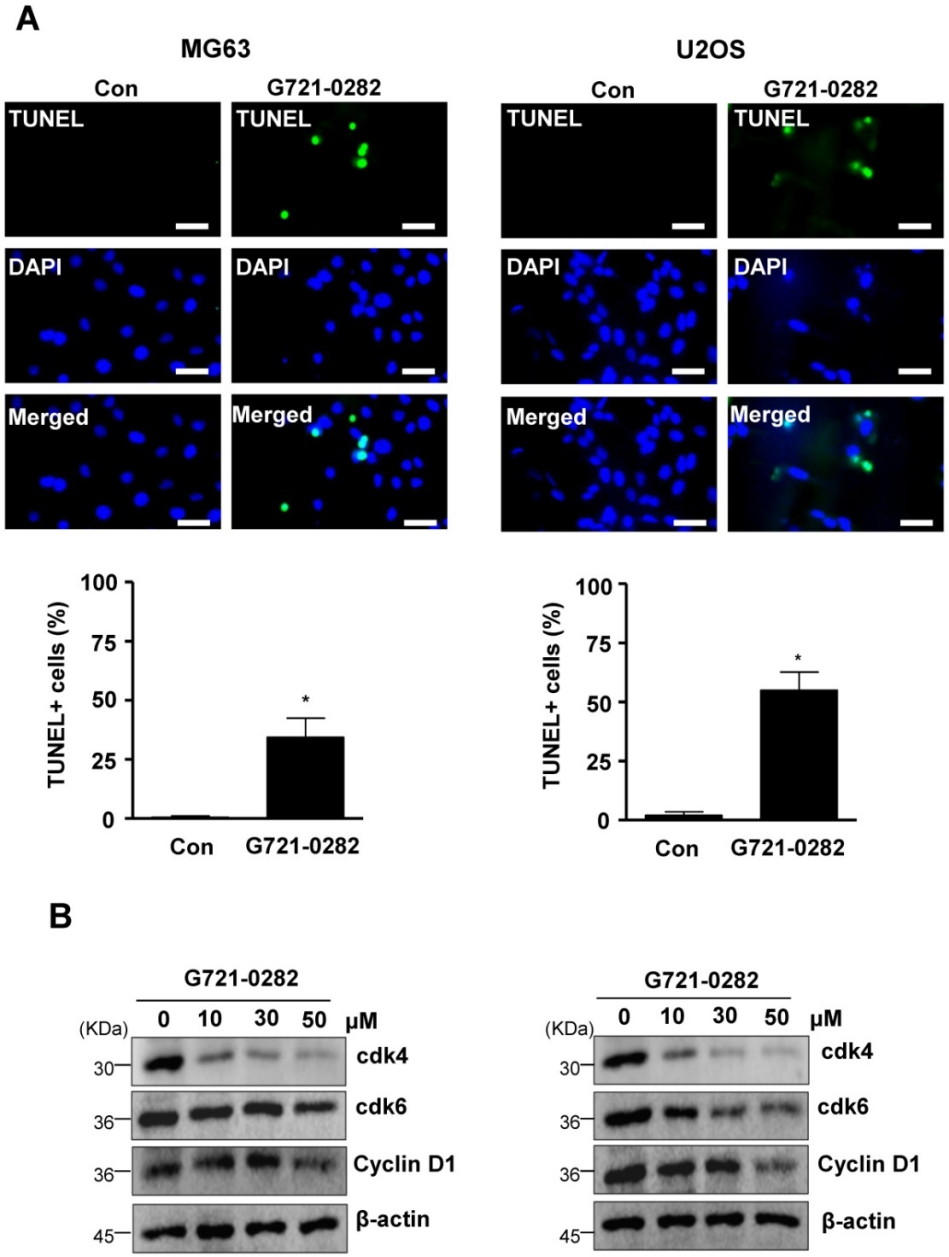
** Effects of G721-0282 on apoptotic cell death in MG63 and U2OS cells. (A)** The cells were treated with G721-0282 for 24 h, and incubated with a FITC-conjugated TUNEL, and analyzed by a confocal microscope (*up*), and TUNEL+ cells (%) were counted in 4 separate locations and expressed as bar graph (*bottom*). **(B)** The equal amounts of cell lysates were analyzed by Western blotting using antibodies against cdk4, cdk6, and Cyclin D1. β-actin was used as a loading control. These data were representative of three independent experiments. Scale bar: 30 μm. *: statistically significant difference compared to the control group (p<0.05).

To further demonstrate the anti-tumor effects of G721-0282, we examined for apoptosis by analyzing DNA strand breaks. Apoptotic cells were also significantly increased by the treatment of G721-0282 as observed by confocal microscopy (Fig. 2A). Cyclin D1, which is required for cell proliferation and for the transition from the G1 to S phase of the cell cycle, is regulated by cdk4 and cdk6. We, next, demonstrated that the treatment of G721-0282 suppressed the expression of Cyclin D1, cdk4, and cdk6 in a dose-dependent manner (Fig. 2B). In addition, these observations were also confirmed by Annexin V assay Supplementary Fig 1.

### G721-0282 regulates apoptotic regulatory proteins and MAPK proteins

**Figure 3:**
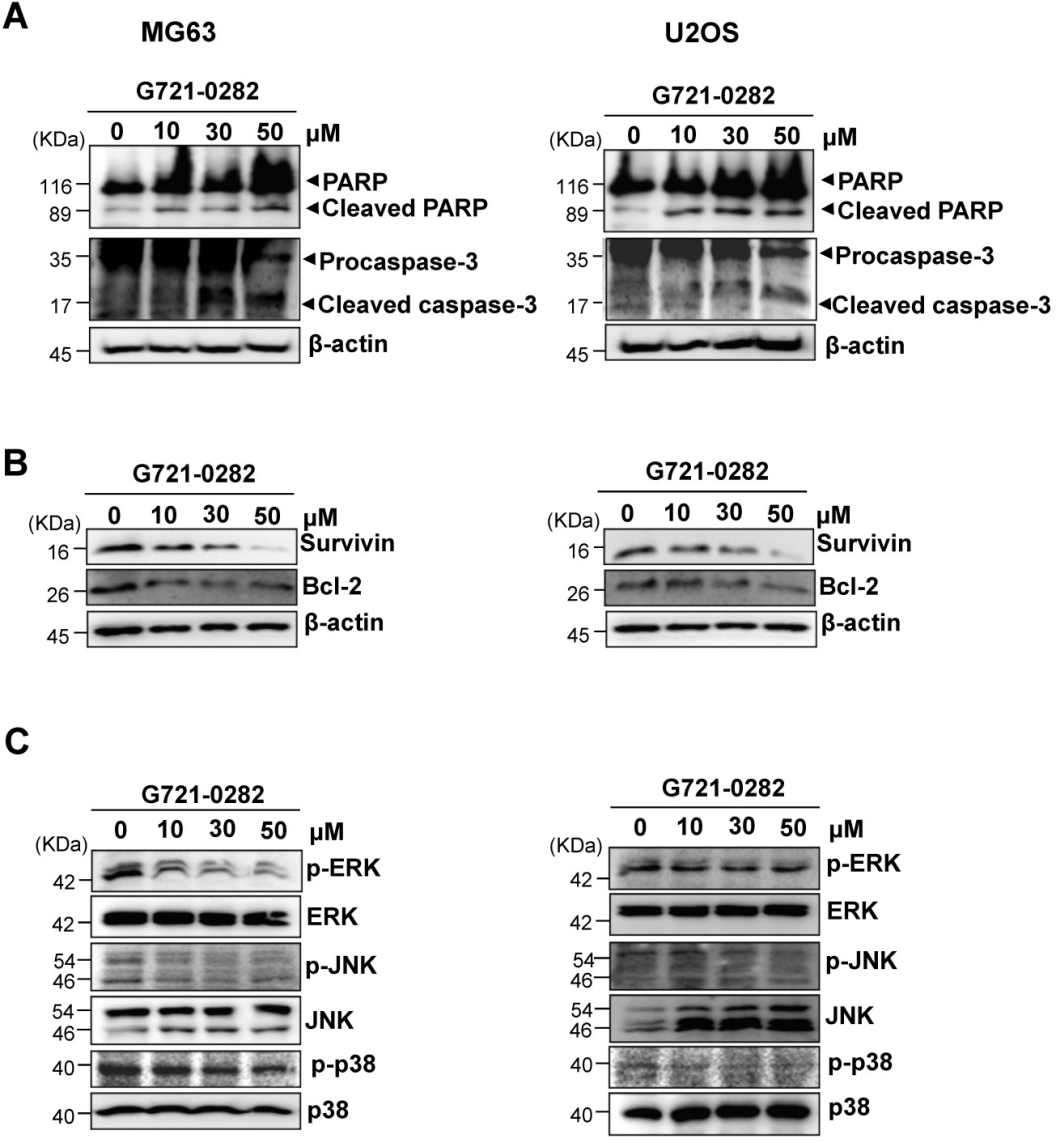
** Effects of G721-0282 on expression of apoptotic regulatory proteins in MG63 and U2OS cells. (A, B)** The cells were treated with the indicated concentration of G721-0282 for 24 h, and then equal amounts of lysates were analyzed by Western blotting using antibodies against PARP and caspase-3 (A), or Survivin and Bcl-2 (B). β-actin was used as a loading control. **(C)** The cells were treated with the indicated concentration of G721-0282 for 1 h, and then equal amounts of lysates were detected with antibodies against phospho-ERK (p-ERK), ERK, phospho-JNK (p-JNK), JNK, phospho-p38 (p-p38), and p38. These data were representative of three independent experiments.

To investigate the mechanisms involved in G721-0282-induced apoptosis, the expression levels of apoptotic and antiapoptotic proteins was analyzed in MG63 and U2OS cells. G721-0282 induced the cleavage of PARP and procaspase-3 as seen by the disappearance of the PARP and procaspase-3 band and appearance of their cleavage products (Fig. 3A). In contrast, G721-0282 inhibited the expression of antiapoptotic proteins such as Survivin and Bcl-2 in a concentration-dependent manner (Fig. 3B). Western blot analysis also showed that treatment of G721-0282 significantly reduced the activation of mitogen-activated protein kinase (MAPK) such as ERK1/2, JNK and p38 MAPK in a dose dependent manner (Fig. 3C).

### G721-0282 inhibits the STAT3 pathways

**Figure 4:**
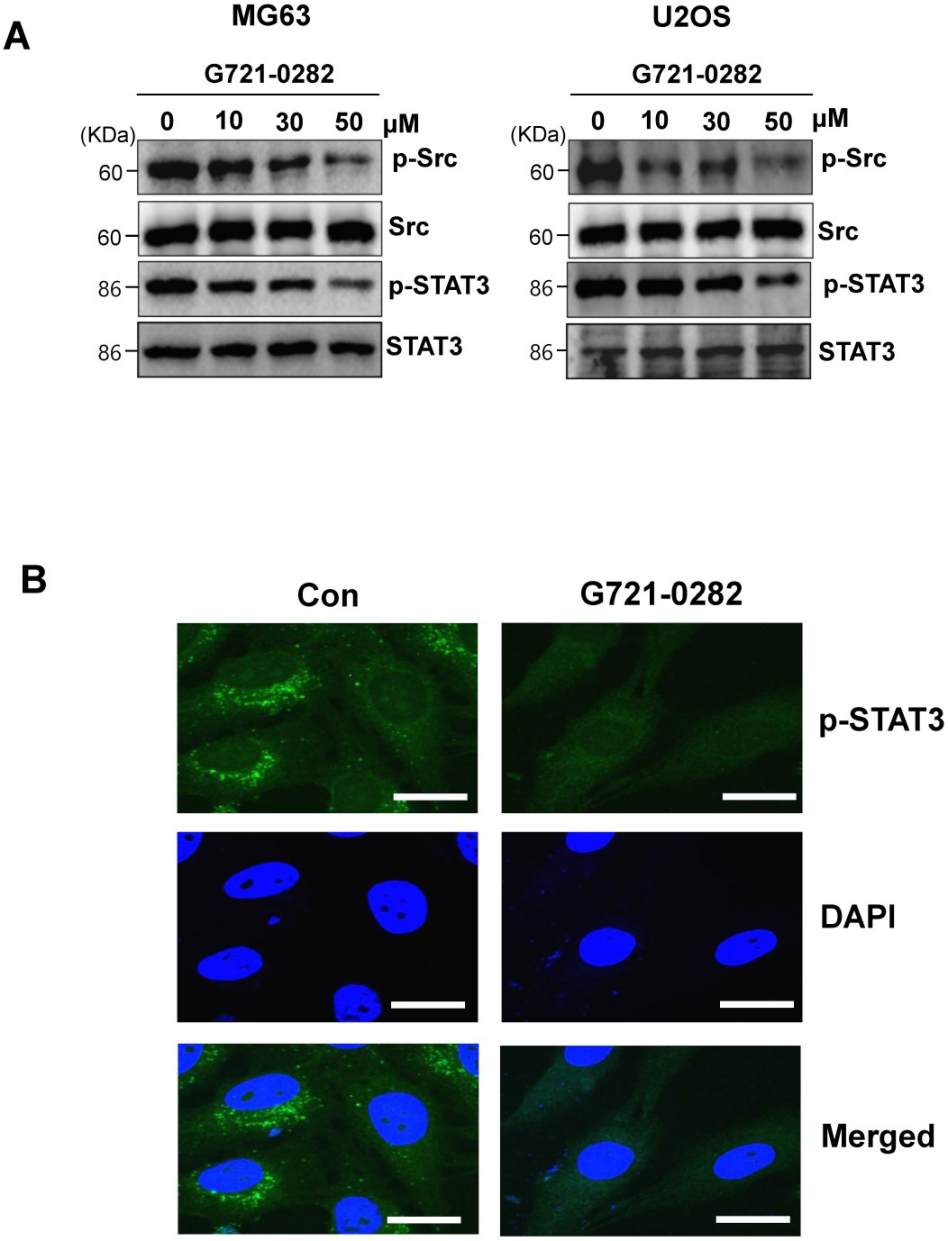
** Effects of G721-0282 on the STAT3 pathway. (A)** The cells were treated with the indicated concentration (10, 30, and 50 μM) of G721-0282 for 6 h. The equal amounts of lysates were analyzed by Western blotting and detected with antibodies against phospho-Src (p-Src), Src, phospho-STAT3 (p-STAT3), and STAT3. **(B)** 6 h after 50 μM G721-0282 treatment, the cells were fixed and permeabilized. p-STAT3 (*green*) was immunostained with rabbit anti-p-STAT3 antibody, followed by Alex-488-conjugated secondary antibody. And then the cells were stained with DAPI (a nuclear marker, *blue*). The *bottom panels* show the merged images of the *first* and* second panels*. Scale bar: 20 μm. These data were representative of three independent experiments.

**Figure 5:**
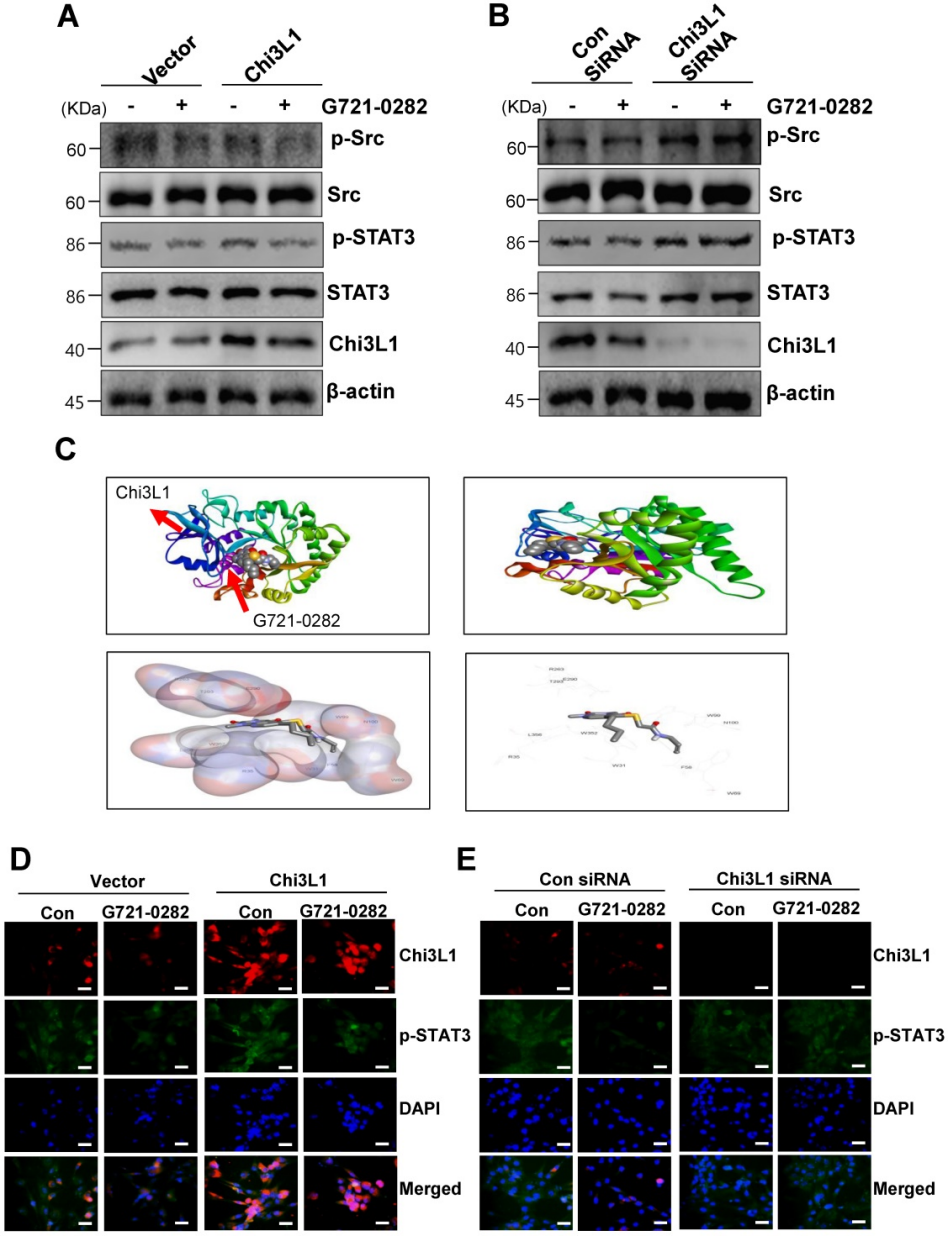
** Effects of Chi3L1 on the G721-0282-mediated STAT3 pathway. (A - B)** The cells were transfected with Chi3L1 plasmid (A) or Chi3L1 siRNA (B) for 24 h. After then, p-Src, Src, p-STAT3, STAT3, Chi3L1, and β-actin were analyzed by Western blotting. **(C)** Molecular surface representation in the docking model of G721-0282 with Chi3L1. **(D - E)** After the cells were transfected with Chi3L1 plasmid (C) or Chi3L1 siRNA (D) for 24 h, the cells were fixed and permeabilized. Chi3L1 (*red*) and p-STAT3 (*green*) was immunostained with anti-Chi3L1 and anti-STAT3 antibodies, followed by Alex-488- and 568-conjugated secondary antibodies. And then sections were stained with DAPI (*blue*). Scale bar: 30 μm. These data were representative of three independent experiments.

MG63 and U2OS cells are known to express constitutively active Src and STAT3. Next, we investigated whether G721-0282 modulates the constitutive Src and STAT3 activation in OS cells. As shown Fig. 4A, G721-0282 inhibited the constitutive activation of Src and STAT3 in a dose-dependent manner (Fig. 4A). In addition, G721-0282 treatment showed that the phosphorylation and translocation of STAT3 in the DAPI-stained nucleus was decreased in MG63 cells. To investigate whether Src and STAT3 were affected by Chi3L1, the cells were transfected with Chi3L1 gene or Chi3L1 siRNA. The inhibitory effect of G721-0282 was increased by the overexpression of Chi3L1, but decreased by the knockdown of Chi3L1 (Fig. 5A-B). To identify the possible binding site of G721-0282 to Chi3L1, we performed computational docking experiments with G721-0282 and Chi3L1 (Fig. 5C). We found that G721-0282 interacts with Chi3L1 on W31, R35, F58, W59, W99, N100, R263, E290, T293, W352, and L356 in the docking model (Fig. 5C). Consistently, the phosphorylation and translocation of STAT3 were confirmed by confocal microscopy (Fig. 5D-E).

### G721-0282 inhibits cell migration, invasion and *in vitro* colony formation

**Figure 6:**
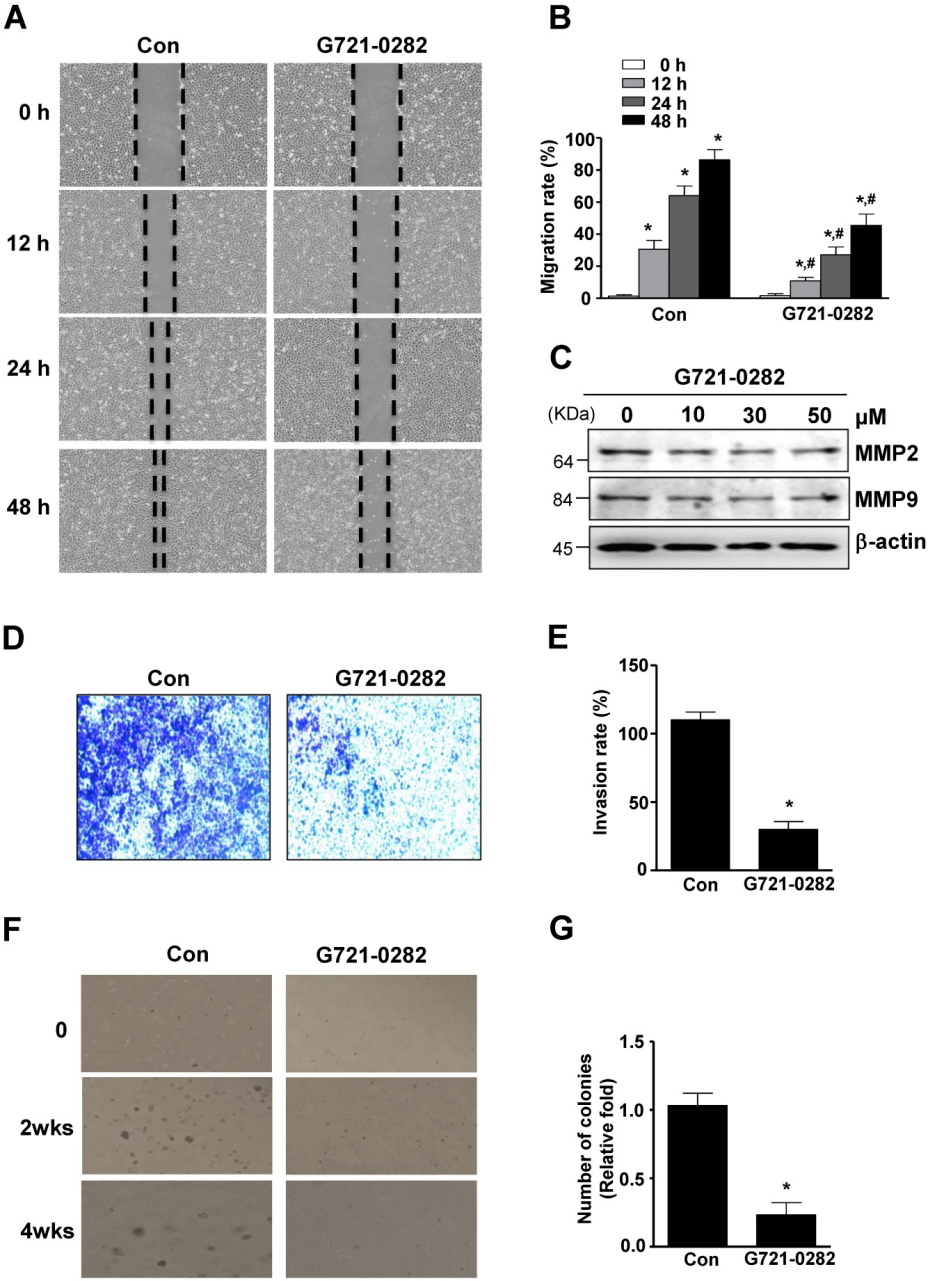
** Effects of G721-0282 on cell migration, invasion, and tumor growth**. **(A - B)** The cells were treated for the indicated time of G721-0282 in MG‐63 cells, and cell migration was observed under a light microscope (A). The migration rate (%) was measured and the bar graph was normalized to control (B). **(C)** 24 h after G721-0282 treatment, equal amounts of lysates were analyzed by Western blotting using antibodies against MMP2 and MMP9. **(D - E)** The boyden chamber assay results showed that more invaded cells were seen in the G721-0282 group than the control group (D), and the bar graph of invasion rate (%) was normalized to control (E). **(F - G)** The cells (1.6 × 10^4^ cells/well) were plated in the top layer and incubated with G721-0282 for 2 and 4 weeks. Colony formation was observed under a light microscope (F) and the colonies were counted. The bar graph was normalized to control (G). These data were representative of three independent experiments. *: statistically significant difference compared to 0 h (p<0.05). #: statistically significant difference compared to the control group (p<0.05).

The anti-migratory effect of G721-0282 on OS cells was evaluated by wound healing migration assay. In MG63 cells, cell migration was significantly suppressed by G721-0282 after 12, 24, and 48 hours of incubation, compared to control (Fig. 6A-B). Since MMPs were involved in extracellular matrix (ECM) degradation, metastasis, and invasion, the expression of MMPs was examined using western blotting. As shown in Fig. 6C, G721-0282 reduced MMP2 and MMP9 levels in a dose-dependent manner (Fig. 6C). Next, the inhibitory effect of G721-0282 on cell invasion was demonstrated by cell penetration through the Matrigel-coated polycarbonate filter in the Boyden chamber. The results showed that G721-0282 suppressed cell invasion, compared with control (Fig. 6D-E). We further investigated the effect of G721-0282 on anchorage-independent growth by soft agar colony formation, which is a good model to study tumorigenicity and is closely associated with the transformed property of cell. G721-0282 caused significant reduction in anchorage-independent colony formation, compared with control (Fig 6F-G).

### G721-0282 inhibits tumorigenesis in *in vivo* xenograft nude mice

**Figure 7:**
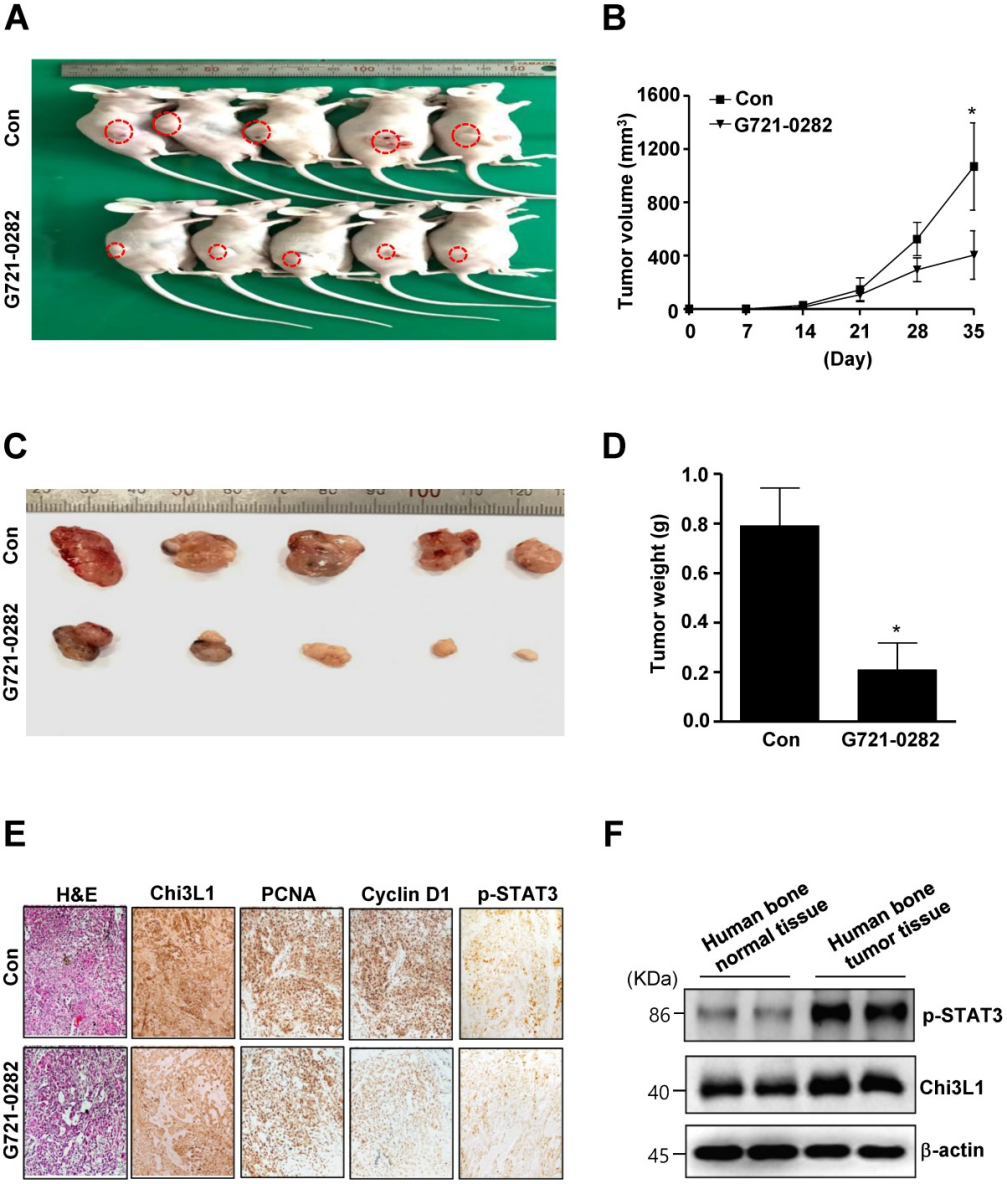
** Effects of G721-0282 on tumor growth in an xenograft mouse model. (A -D)** MG63 cells were injected into the right lower flanks in BALB/c athymic nude mice. All data indicate tumors formed on the right lower flanks. Images of MG63 bearing mice (A) and tumor volume (B), size (C), and weight (D). **(E)** Tumor sections of control and G721-0282-treated MG63 bearing mice were analyzed by H&E stain, and the expression of proteins were analyzed by immunohistochemistry. **(F)** Human bone control and human bone tumor samples were assessed by western blot analysis against p-STAT3, Chi3L1, and β-actin. These data were representative of three independent experiments. *: statistically significant difference compared to the control group (p<0.05).

To verify direct roles of G721-0282 in OS, we investigated the anti-tumor activities of G721-0282 in *in vivo* xenograft model. Tumor development was monitored for 35 days. All mice were killed at the end of the experiment when tumors were dissected and weighted. There was a significant difference in tumor growth between control and G721-0282-treated mice. Representative tumors in the xenograft mice treated with or without G721-0282 are shown in Fig. 7A. The tumor volume (Fig. 7B) and weight (Fig. 7C-D) were significantly decreased compared with the control. Immunohistochemistry (IHC) analysis revealed that significant reduced expression of Chi3L1 and Proliferating cell nuclear antigen (PCNA), Cyclin D1, and p-STAT3 (Fig. 7E). In addition, we demonstrated that Chi3L1 expression was correlated with p-STAT3 level in human bone tumor tissues (Fig. 7F). Overall, our data suggest that G721-0282 suppresses tumorigenesis in tumor development.

## Discussion

OS is considered as the most common type of primary malignant bone tumor, which typically is common in children and adolescents. Its pathogenesis is not yet fully understood, and there is an urgent need for more effective treatment 1, 3. To date, no data have been reported about the effects of G721-0282, a potential ligand of Chi3L1 on OS cells. To investigate the effects of G721-0282, we examined the effects of G721-0282 in commonly used OS cell lines, MG63 and U2OS. MTT and apoptosis assays demonstrated that G721-0282 inhibited the proliferation and induced apoptosis of OS cells in a dose-dependent manner. The results suggest a consequence of apoptosis caused by G721-0282 in OS cells. These results were supported by G721-0282 treatment by upregulation of apoptotic proteins such as cleaved PARP and cleaved caspase-3, but downregulation of anti-apoptotic proteins such as Survivin and Bcl-2. Cyclin D1 was well known for its important role in cell cycle progression through regulating the transition from G1 to S phase and G2 to M phase progression, as well as to mitosis 26. In the present study, we found that the expression of cyclinD1, cdk4, and cdk6 was reduced in MG63 and U2OS, thus our data suggest that G721-0282 has beneficial effects on the inhibitive activities of cell growth in OS.

Several studies have shown that the activation of STAT3 contributes to tumor development including bone, breast, prostate, skin, ovary, blood, and lung 27-29. The pathway plays critical roles in cell proliferation, survival, apoptosis, metastasis, and invasion 30, resulting in the expression of downstream genes including Bcl-2, Survivin, and Cyclin D1 31. Thus, the STAT3 pathway is an important therapeutic target for cancer treatment. Our previous studies demonstrated that bee venom and melittin inhibited the STAT3 pathway and induced the cell growth inhibition of lung cancer 32. It was also reported that activated STAT3 is a critical downstream effector of Src kinanse-induced oncogenic signaling in human cancer cells 13. In the present study, G721-0282 inhibited the phosphorylation of Src kinase and the phosphorylation of STAT3. Furthemore, G721-0282 reduced the expression of the STAT3 target proteins, Bcl-2 and Survivin. These results indicate that the STAT3 pathway is a novel target for the anti-tumor effect of G721-0282.

Reduction in cell adhesion, increased cell migration, intracellular matrix degradation, and invasion to other tissues are correlated with tumorigenesis 4. Matrix metalloproteinases (MMPs) are the best well known enzymes. Among MMPs, MMP2 and MMP9 have pivotal role in angiogenesis, tumor proliferation, and invasion 33. Thus, MMP2 and MMP9 are targets in developing chemotherapeutic drugs and preventing tumorigenesis 34. In the present study, G721-0282 reduced migration, invasion, anchorage-independent growth of OS cells, and inhibited tumor growth in a xenograft nude mouse model. Concomitant with the results, we also observed the decreased tumor proliferation by Chi3L1 and PCNA immunostaining. Furthermore, immunohistochemical analysis confirmed the downregulation of p-STAT3 and Cyclin D1 following treatment with G721-0282 in *vivo*, which was consistent with our findings *in vitro*.

In conclusion, the present study is the first report that G721-0282 shows anti-cancer activity *in vitro* and *in vivo* in OS by inhibiting the STAT3 pathway. These data suggest that G721-0282 may be potentially beneficial for therapeutic effects of OS via the inactivation of STAT3 pathway.

## Supplementary Material

Supplementary figures and tables.
