# Supplementary material for: G721-0282 inhibits cell growth and induces apoptosis in human osteosarcoma through down-regulation of the STAT3 pathway
Source: Int J Biol Sci. 2020 Jan 1;16(2):330–41. doi: 10.7150/ijbs.37781 (PMC6949149; doi:10.7150/ijbs.37781)

- **Supplementary Figure 1. Effects of G721-0282 in Annexin V assay.** The cells were treated with G721-0282 for 24 h, and incubated with a FITC-conjugated annexin V antibody, and analyzed by a confocal microscope Scale bar: 30 μm.

Supplementary F1

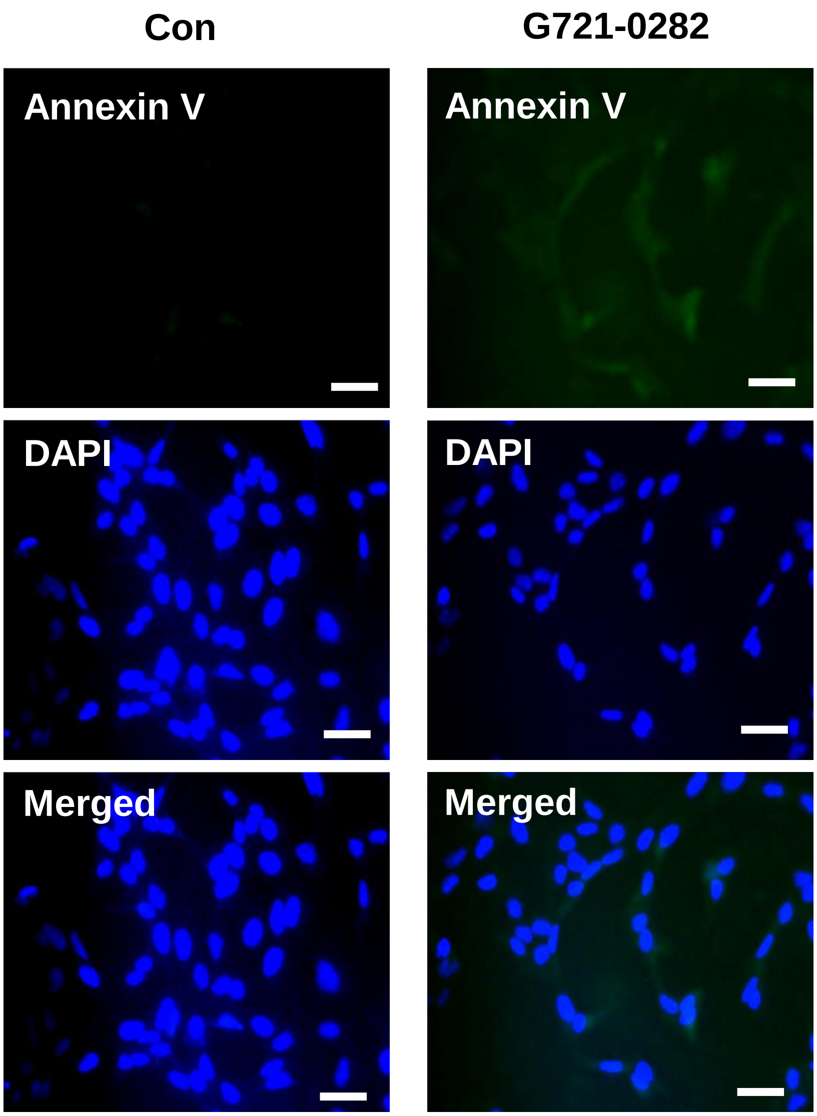

Supplement: Supplementary file 1 — Supplementary figures and tables. [file ijbsv16p0330s1.pdf]
